# Mechanisms by Which Low Glucose Enhances the Cytotoxicity of Metformin to Cancer Cells Both *In Vitro* and *In Vivo*

**DOI:** 10.1371/journal.pone.0108444

**Published:** 2014-09-25

**Authors:** Yongxian Zhuang, Daniel K. Chan, Allison B. Haugrud, W. Keith Miskimins

**Affiliations:** 1 Cancer Biology Research Center, Sanford Research/USD, Sioux Falls, South Dakota, United States of America; 2 Sanford School of Medicine, The University of South Dakota, Vermillion, South Dakota, United States of America; Mayo Clinic College of Medicine, United States of America

## Abstract

Different cancer cells exhibit altered sensitivity to metformin treatment. Recent studies suggest these findings may be due in part to the common cell culture practice of utilizing high glucose, and when glucose is lowered, metformin becomes increasingly cytotoxic to cancer cells. In low glucose conditions ranging from 0 to 5 mM, metformin was cytotoxic to breast cancer cell lines MCF7, MDAMB231 and SKBR3, and ovarian cancer cell lines OVCAR3, and PA-1. MDAMB231 and SKBR3 were previously shown to be resistant to metformin in normal high glucose medium. When glucose was increased to 10 mM or above, all of these cell lines become less responsive to metformin treatment. Metformin treatment significantly reduced ATP levels in cells incubated in media with low glucose (2.5 mM), high fructose (25 mM) or galactose (25 mM). Reductions in ATP levels were not observed with high glucose (25 mM). This was compensated by enhanced glycolysis through activation of AMPK when oxidative phosphorylation was inhibited by metformin. However, enhanced glycolysis was either diminished or abolished by replacing 25 mM glucose with 2.5 mM glucose, 25 mM fructose or 25 mM galactose. These findings suggest that lowering glucose potentiates metformin induced cell death by reducing metformin stimulated glycolysis. Additionally, under low glucose conditions metformin significantly decreased phosphorylation of AKT and various targets of mTOR, while phospho-AMPK was not significantly altered. Thus inhibition of mTOR signaling appears to be independent of AMPK activation. Further in vivo studies using the 4T1 breast cancer mouse model confirmed that metformin inhibition of tumor growth was enhanced when serum glucose levels were reduced via low carbohydrate ketogenic diets. The data support a model in which metformin treatment of cancer cells in low glucose medium leads to cell death by decreasing ATP production and inhibition of survival signaling pathways. The enhanced cytotoxicity of metformin against cancer cells was observed both in vitro and in vivo.

## Introduction

In the transformation to cancer, cells undergo reprogramming of their ordinary metabolic functions to facilitate rapid growth potential. Otto Warburg reported high rates of glycolysis in cancer cells even in aerobic conditions. This paradoxical change is one mechanism by which cancer cells have adapted for rapid proliferation. As a result of this altered metabolism, cancer cells use large amounts of glucose and generate high amounts of lactate. Glucose metabolism through glycolysis contributes to ATP synthesis and provides intermediates for other biosynthetic processes. Thus, cancer cells are dependent on the high rates of glucose uptake and metabolism for survival [1], [2].

Current methods of *in vitro* cancer cell culture commonly use high glucose, 25 mM (450 mg/dL), in the growth medium. While high glucose medium creates an optimal environment for cancer cell proliferation, these glucose levels may complicate the interpretation of drug efficacy studies. High glucose alone has the ability to activate proliferation pathways in a cancer cell [2], and the constant availability of glucose places little of the normal stress cancer cells experience *in vivo*. In pancreatic cancer cells, Sinnett-Smith *et al*. [3] found that metformin’s actions on AMPK activation were enhanced through use of 5 mM glucose media in cultured cells.

Normal serum glucose is usually maintained between 4 and 6 mM (approximately 72–108 mg/dL). In cases of low nutrient availability, serum glucose levels may drop to 2.5 mM (45 mg/dL), with tissue levels of glucose commonly lower. Reducing glucose availability has also been attempted as a cancer treatment by a variety of methods. Modifying diet by direct caloric restriction or fasting has been investigated as a method of reducing cancer growth with some promising results [4]–[6]. Generally, fasting appears to be more effective than constant caloric restriction at reducing cancer growth [5]. Besides diet modification, anti-diabetic drugs are another means of lowering plasma glucose. In a clinical trial comparing metformin’s efficacy to existing type II diabetes medications, metformin was found to lower plasma glucose concentrations by approximately 3 mM (55 mg/dL) from pretreatment levels [7]. However, it is worth noting that metformin did not have significant effects on reduction of plasma glucose in non-diabetics [8].

Recently metformin has gained renewed interest as a potential cancer therapeutic and chemotherapy adjuvant. Metformin’s potential anti-cancer activity was implicated by the lower incidence of cancer in type II diabetics versus other glucose controlling drugs [9]. This effect was initially attributed to metformin’s systemic glucose and insulin regulating properties. Later metformin was also found to inhibit cancer growth at the cellular level. The anti-oncogenic action for metformin is likely a combination of systemic insulin control effects and direct cellular effects. Many groups have demonstrated metformin’s action *in vitro* in a variety of cancer types [10]–[14]. However, to mimic the effects of metformin *in vitro* that are observed *in vivo*, high concentrations, commonly 5–20 mM, of metformin are necessary. Recent studies suggest these findings may be due in part to the common cell culture practice of utilizing high glucose, and when glucose is lowered, metformin becomes increasingly cytotoxic to cancer cells [15], [16]. Additionally, the carbon source for cancer cells was found to significantly alter anti-cancer effects of metformin when comparing glucose to glutamine [17]. In this study cancer cells exposed to high glutamine in the absence of glucose were much more sensitive to inhibition by metformin.

While the precise mechanism for metformin’s cancer cytotoxicity remains unidentified, metformin’s systemic actions likely combine with the direct cellular action to enhance its effect on cancer cell growth inhibition and cancer cell death. Here we show that low to normal glucose levels, as opposed to high glucose conditions, potentiate the effect of metformin in breast and ovarian cancer cell lines. The possible mechanisms for this activity are explored. These observations may reveal both more relevant cell culture techniques for studying metformin in cancer as well as provide insight into clinical use of metformin as an adjuvant cancer therapy.

## Methods

### Chemicals and reagents

The following chemicals were used in this study: metformin (1, 1-dimethylbiguanide,), D-(−)-fructose, D-(+)-galactose, 2-deoxy-D-glucose, oligomycin (Sigma Chemical Co). Dulbecco’s modified Eagle’s Medium (DMEM) with high glucose (Hyclone), Gibco DMEM without glucose (Life Technology), SYTOX Green Nucleic Acid Stain (Invitrogen), and ATP Assay kit (Invitrogen).

### Cell culture

Human cancer cell lines MCF7, MDAMB231, OVCAR3, PA-1, SKRB3, and human mammary epithelial MCF10A cells were all purchased from ATCC and maintained in DMEM containing 10% fetal bovine serum with varying glucose levels and 1% penicillin-streptomycin at 37°C under a humidified atmosphere containing 5% CO_2_. All the cell lines were used within passage ten after receiving from ATCC to ensure cell line authentication.

### Cell death assay using Sytox Green Nucleic Acid Stain

Cells were plated into 96 well plates and treated with the indicated treatment for one or two days. Sytox Green nucleic acid stain (10 µM) was added directly to cells in 96 well plates and incubated for 10 minutes. The plate was read at excitation/emission of 485 nm and 530 nm, respectively, with a 515 nm cutoff using a fluorescence plate reader (SpectraMax M5 Multi-Mode Microplate Reader, Molecular Devices, LLC). Fluorescence was measured to obtain number of dead cells. Subsequently, to determine total cell number, 0.4% Triton-X100 was added to each sample and it was incubated for 30 minutes at room temperature to permeabilize all the cells, and fluorescence at 485/530 nm was measured again to obtain the total cell number.

### ATP assay

Cells were seeded into 6 well plates followed by incubation for 24 hours. Treatment was given with fresh medium for 24 hours. Equal numbers of cells were lysed in each treatment group and 10 µl was used from each sample following the manufacturer’s protocol for ATP assays (Invitrogen, ATP Determination Kit, A22066). Briefly, 100 µl of the standard reaction solution was measured in a luminometer for background luminescence. Then 10 µl of the lysate supernatant was added to the reaction solution and the luminescence was again measured. Background luminescence was subtracted from sample luminescence and results were plotted as fold change from control samples.

### Western blotting

Cells in 35 mm dishes were rinsed once with PBS and lysed by addition of sodium dodecylsulfate (SDS) sample buffer [2.5 mM Tris-HCl (pH 6.8), 2.5% SDS, 100 mM dithiothreitol, 10% glycerol, 0.025% pyronine Y]. Equal amounts of protein from each treatment group were separated on 10% or 15% SDS-polyacrylamide gels. Proteins were transferred to Immobilon P membranes (Millipore) using a semi-dry Bio-Rad Trans-blot apparatus with a transfer buffer of 48 mM Tris-HCl and 39 mM glycine. The membranes were blocked with 5% non-fat dry milk or 5% BSA in Tris-buffered saline [10 mM Tris-HCl (pH 7.5), 150 mM NaCl] containing 0.1% Tween-20 (TBS-T) for one hour at room temperature. The membrane was then incubated with the appropriate antibody in TBS-T containing 5% non-fat dry milk or 5% BSA for 1 hour at room temperature or overnight at 4°C. After washing in TBS-T the membrane was incubated with the appropriate horseradish peroxidase (HRP)-conjugated secondary antibody. Proteins were detected using the Super Signal West Pico chemiluminescent substrate (Pierce Biochemical). Anti-β-actin monoclonal antibody (A5441, used at 1∶10,000) was purchased from Sigma. Antibodies against phosphorylated AKT at threonine 473 (#05-669 used at 1∶1000) was purchased from Upstate Biotechnologies. Antibodies against AKT phosphorylated at threonine 308 (#4056, used at 1∶1000), ATK (#4691, used at 1∶1000), phosphorylated S6K (#9206, used at 1∶2000), S6K (#9202, used at 1∶2000), PARP (#9542, used at 1∶2000), cleaved PARP (#9541, used at 1∶2000), AMPK phosphorylated at threonine 172 (#2535, used at 1∶1000), and AMPK (#2532, used at 1∶1000), Cleaved Caspase 7 (#9491, used at 1∶1000) were purchased from Cell Signalling Technology. Secondary horseradish peroxidase-linked anti-mouse (#31430, used at 1∶5000) and anti-rabbit (#31460, used at 1∶5000) IgG antibodies were purchased from Pierce Biochemical.

### Lactate assay

MCF7 were plated in 96 well plates and treated as indicated for 15 hours. Medium from each treatment was collected and tested for lactate concentration using an L-Lactate Assay Kit (Eton Biosciences Inc.). Cells were counted using Sytox Green staining method as described previously. Relative lactate levels were obtained after normalizing by total cell number.

### Measurement of Extra Cellular Acidification Rate (ECAR) and Oxygen Consumption Rate (OCR)

ECAR and OCR were measured using a Seahorse XF24 analyzer according to manufacturer’s instructions (Seahorse Bioscience). Briefly, MCF7 cells were plated at 40,000 cells/well in XF24-well plates. Cells were treated as indicated the next day. Before being processed with the Seahorse XF24 analyzer, cells were washed and equilibrated with buffer free medium (D5030, Sigma) at 37°C in a CO_2_-free incubator for one hour. Initial measurements of ECAR and OCR were obtained followed by addition of different concentrations of glucose (2.5 mM or 25 mM), fructose (25 mM) or galactose (25 mM). ECAR and OCR were further measured following injection of oligomycin and 2-deoxyglucose. Cell number was obtained by trypsinizing cells and counting using a hemocytometer. ECAR and OCR were plotted after normalizing by total cell number.

### *In vivo* mouse studies

All experiments were performed in accord with institutional and national guidelines and regulations; the protocol was approved by the institutional animal care and use committee at Sanford Research. Briefly, using a 25-gauge needle, Balb/C mice were subcutaneously injected with 1×10^5^ cells in the flank (10 mice per treatment condition). Four days after tumor cell injection, the mice were switched to a calorie restricted low carbohydrate ketogenic diet (BioServ P3666) for the KD group (see below for details of diets). The control groups remained on normal mouse chow (Teklad Global 18% Protein Rodent Diet) and were fed as libitum. Metformin (2 mg/mouse) was administered intraperitoneally daily starting 7 days after tumor injection. Tumor volume was estimated using A^2^×B where A is the larger diameter and B is the smaller diameter. Animals were euthanized when the tumor size was greater than 15 mm in its greatest dimension, or tumor volumes reach 3000 mm^3^, or when the animal was substantially emaciated.

### Serum glucose measurement

Serum glucose levels were measured using tail vein blood after puncturing the tail with a 25 gauge needle to produce one drop of blood for each test. Glucose was measured using a Bayer Contour Glucometer and glucose test strips.

### Diet information

The low carbohydrate ketogenic diet was purchased from BioServ (#F3666). This diet provides calories from protein, fat and carbohydrates at approximately 4.6%, 93.4%, and 2%, respectively. All mice being fed this diet were subjected to 30% calorie restriction (7 kCal/day/mouse) starting the 4^th^ day after tumor cell injection. Calorie restriction was determined by measuring the weight of food consumed each day. The appropriate amount of the ketogenic diet was provided every day on a petri dish within the cage. Calories were increased to 7.5 kCal/day/mouse (25% restriction) at day 7 after initiation of the ketogenic diet to prevent weight loss. Calories were further increased to 8 kCal/day/mouse at day 11 after the initiation of ketogenic diet and maintained at this level until the end of the experiment. The control diet groups were fed ad libitum on standard mouse chow (Teklad Global 18% protein rodent diet) which provides calories from protein, fat and carbohydrates at approximately 24%, 18%, and 58%, respectively.

### Statistical analysis

Error bars shown are standard deviations from the mean of at least three replicates. Two-tailed pairwise Student’s *t* tests were used to compare two groups. *P* values less than or equal to 0.05 were considered to have significance.

## Results

**Figure 1:**
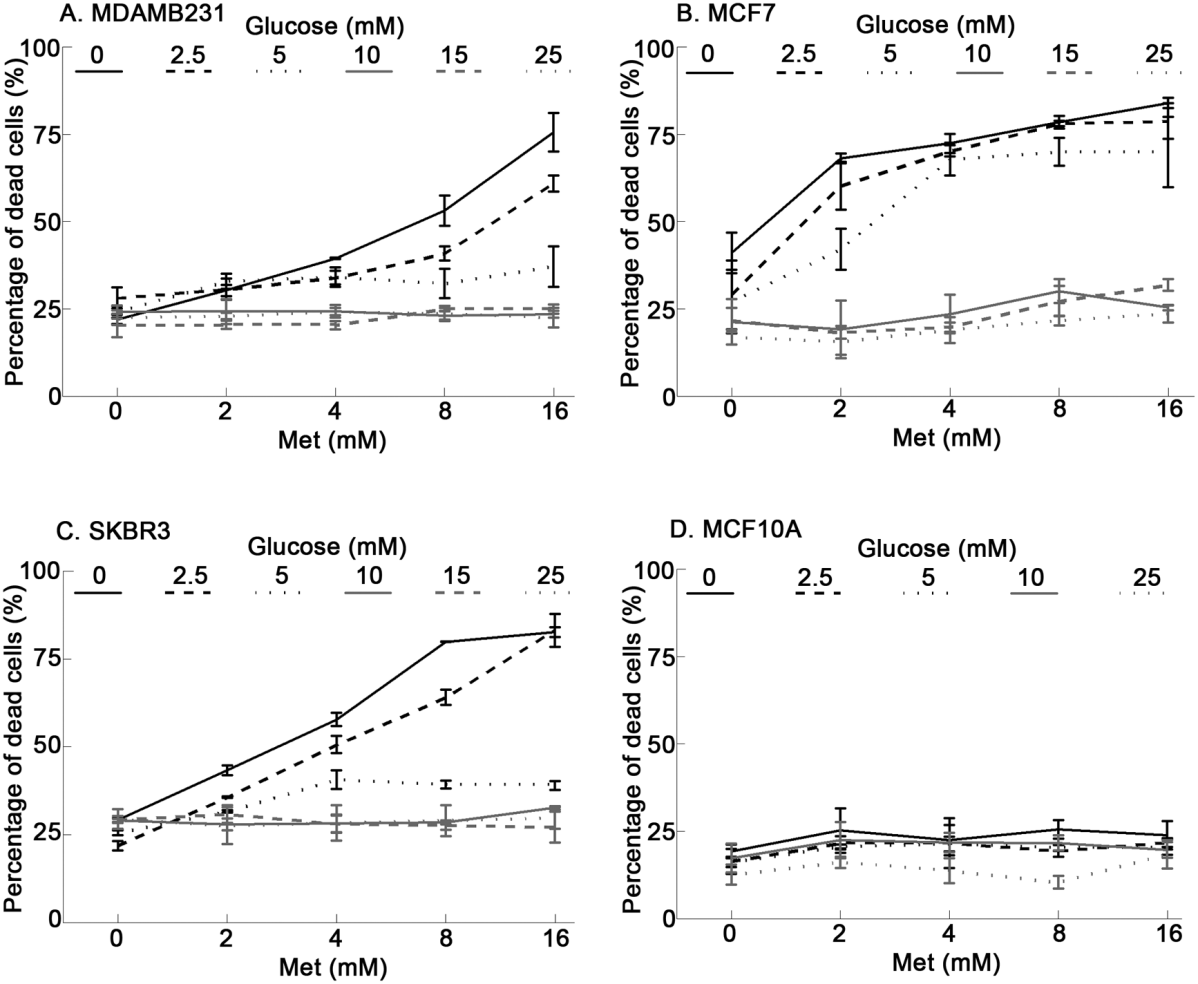
Reduction of glucose in the culture medium sensitizes breast cancer cells to metformin treatment but not human mammary epithelial MCF10A cells. All cells were treated with different concentrations of metformin (0, 2, 4, 8, 16 mM) in medium containing different levels of glucose (0, 2.5, 5, 10, 15, 25 mM) for one day. Percentage of dead cells was determined using Sytox Green staining. Metformin significantly increased percentage of dead cells with decreasing glucose concentration in (**A**) MDAMB231 cells, (**B**) MCF7 cells, and (**C**) SKBR3 cells but not in (**D**) MCF10A cells. Data were presented as mean ± standard deviation.

Our previous research has demonstrated that metformin is cytotoxic to many breast and ovarian cancer cell lines. However, some cell lines such as the breast cell lines MDAMB231 and SKBR3 exhibited resistance to metformin cytotoxicity [14], [18], [19]. Previous experiments were carried out in DMEM containing 25 mM glucose, which is significantly higher than normal physiological conditions of 4–8 mM. To determine the influence glucose concentration has on the cytotoxicity of metformin, we tested the effects of different concentrations of glucose in the culture medium of cancer cells exposed to metformin treatment. All cell cultures were initially maintained in high glucose medium (25 mM glucose) and plated into 96 well plates for one day, the next day cells were treated with metformin (0 mM, 2 mM, 4 mM, 8 mM and 16 mM) in medium containing different concentrations of glucose (0 mM, 2.5 mM, 5 mM, 10 mM, 15 mM and 25 mM) for one day. As glucose levels in the medium decrease, metformin treatment significantly increased the percentage of dead cells in MDAMB231, MCF7, and SKBR3 cells (Figure 1A, 1B, 1C). Previous data show that, in high glucose medium, both MDAMB231 and SKBR3 cells are resistant to metformin treatment (8 mM) for up to three days [14], [18], [19]. In high glucose conditions these cell lines, MDAMB231 and SKBR3, continued to be resistant to metformin treatments up to 16 mM concentrations of the drug. However, when the glucose levels were reduced to 2.5 mM or less both MDAMB231 and SKBR3 show a cytotoxic response to metformin treatment from 4 mM to 16 mM.

Concurrently, the effects of metformin at different concentrations of glucose were examined in the non-cancer human mammary epithelial cell line MCF10A (Figure 1D). In contrast to the cancer cell cultures, reduction of glucose concentration did not significantly sensitize MCF10A to metformin treatment over this time period. This difference may be a result of underlying differences in glucose metabolism between normal mammary epithelial cells and cancer cells.

**Figure 2:**
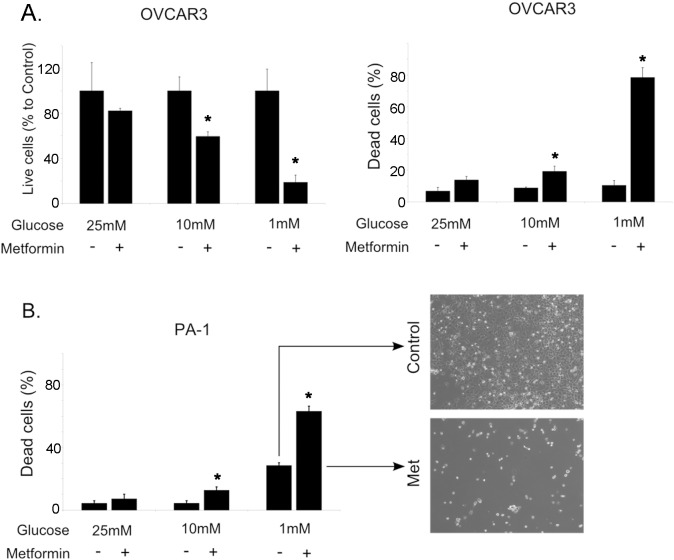
Metformin treatment of ovarian cancer cells is enhanced by low glucose conditions. **A.** After 48 hour treatments with metformin (5 mM, +) or control vehicle (H_2_O, –), live OVCAR3 cell number was determined by counting trypan blue negative cells and dead cell number was determined by counting trypan blue positive cells. **B.** PA-1 cell death was determined as described in A. Phase contrast images (40x) of the cells cultured with (lower image) or without (upper image) metformin treatment. Bar graphs represented mean ± standard deviation. All metformin treated groups in medium containing low glucose (1 mM) were significantly different from their control groups. *Indicates significant difference between groups.

In order to test whether these effects on metformin cytotoxicity applied to other cancer cell types, we also examined ovarian cancer cells. In a similar fashion, lowering the glucose was found to increase the cytotoxicity of metformin in the ovarian cancer cell lines OVCAR3 and PA-1 (Figure 2). This suggests that lowering the glucose flux in cancer cells could significantly enhance the cytotoxicity of metformin, and that this effect may be broadly relevant to various cancer cell types.

**Figure 3:**
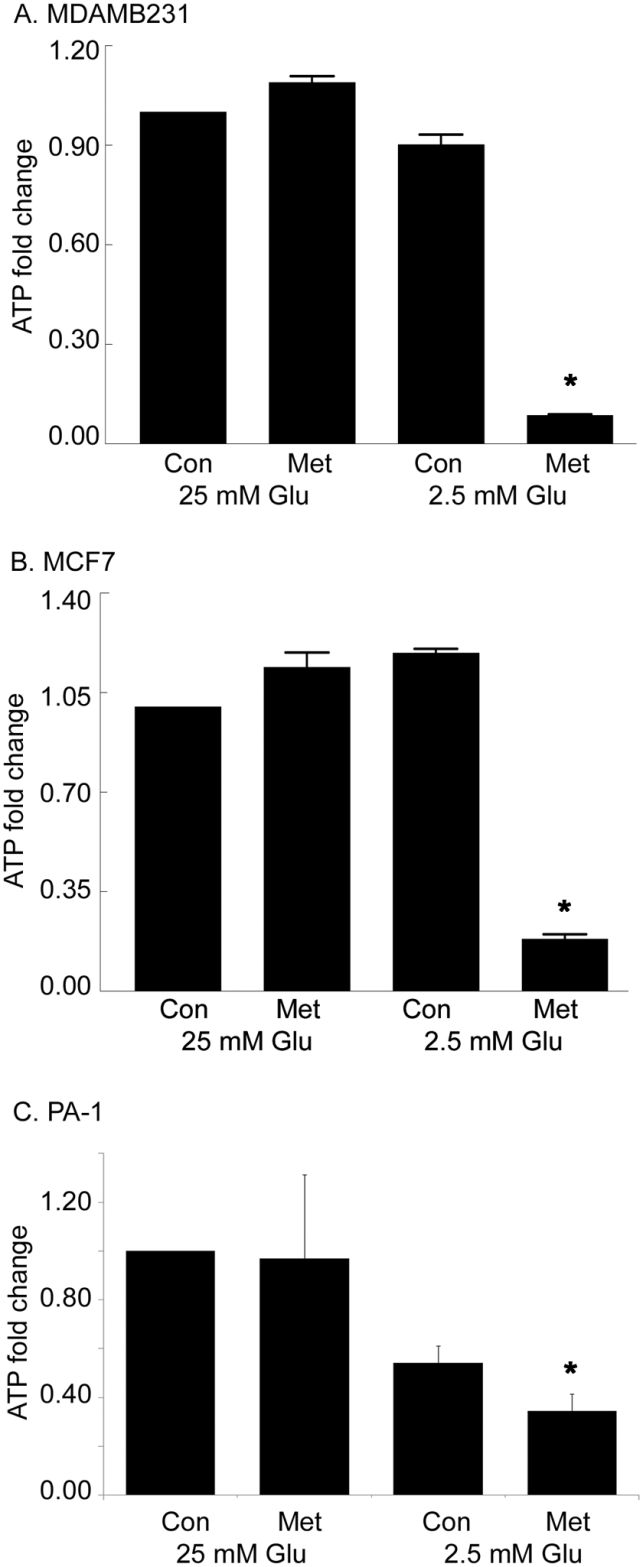
Metformin decreases ATP levels in medium containing low glucose (2.5 mM). MDAMB231 (**A**) and (**B**) MCF7 cells were treated with metformin (8 mM) in either 25 mM glucose or 2.5 mM glucose for one day and ATP levels were measured and fold change of ATP compared with control was plotted. **C.** ATP measurements in PA-1 cells after 12 hours with or without metformin. This time point was chosen because of the rapid rate of PA-1 growth under control culture conditions. Data were presented as mean ± standard deviation. All metformin treated groups in medium containing low glucose (2.5 mM) were significantly different from their control groups. *Indicates significant difference between groups.

Other known cellular actions of metformin include inhibition of complex I of the electron transport chain and oxidative phosphorylation [20]. This action can result in deceased production of ATP and other cellular intermediates. The cellular ATP levels of MCF7, MDAMB231, and PA-1 were examined after metformin treatment (24 hours) in either high glucose (25 mM) or low glucose (2.5 mM) tissue culture medium (Figure 3). The results show that metformin strongly decreases ATP levels only in low glucose medium. In high glucose conditions, at this time point, metformin tended to increase ATP but not to a level of significance.

**Figure 4:**
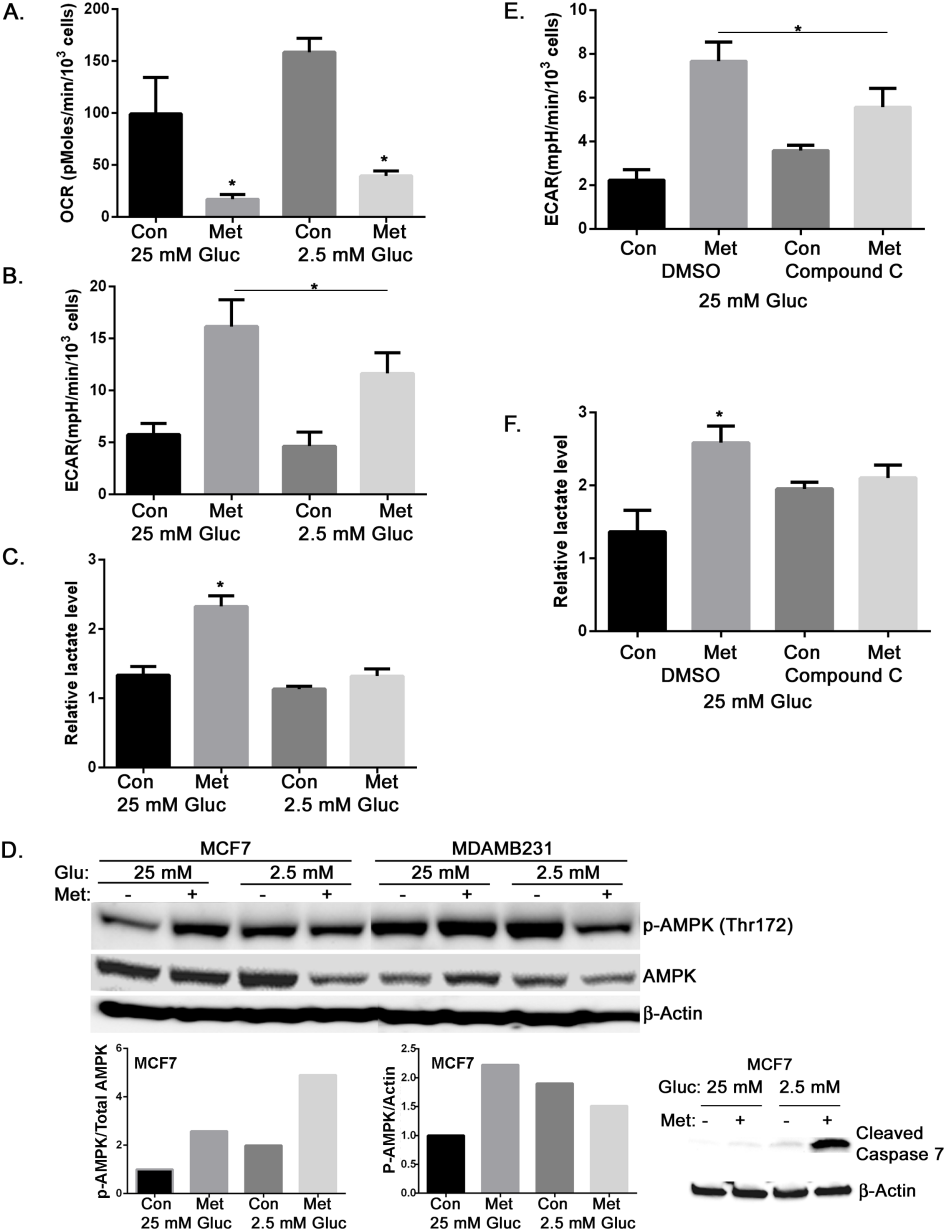
Metformin inhibits oxidative phosphorylation and increases glycolysis in 25 mM glucose containing medium in an AMPK-dependent manner. **A.** MCF7 cells were treated with metformin (8 mM) for 15 hours in either 25 mM or 2.5 mM glucose containing media. Oxygen consumption rate (OCR) was determined using the FX24 instrument for metabolic flux analysis. Metformin treated groups were significantly different from their control groups. **B.** MCF7 cells were treated with metformin (8 mM) for 15 hours. Extracellular acidification rate (ECAR) was determined using the FX24 instrument for metabolic flux analysis. Metformin treated groups were significantly different from each other and their control groups. **C.** MCF7 cells were treated with metformin (8 mM) for 15 hours, and medium lactate levels were measured as an indicator of glycolytic flux. Metformin treated groups were significantly different from each other. **D.** Extracts of MCF7 and MDAMB231 cells harvested one day treatment with metformin in either 25 or 2.5 mM glucose were used for Western blotting detection of phosphorylated AMPK and total AMPK. β-Actin was detected as a loading control. Densitometry of p-AMPK/AMPK or p-AMPK/Actin for MCF7 cells is presented as a bar graph. Cleaved capsase 7 in MCF7 cells was detected with western blotting. β-Actin was detected as a loading control. **E.** MCF7 cells in high glucose were treated with metformin (8 mM) and compound C (10 µM) as indicated for 15 hours. DMSO is the vehicle control for compound C. ECAR was determined as described in B. Metformin treated groups were significantly different from each other. **F.** MCF7 cells in high glucose were treated with metformin (8 mM) and compound C (10 µM) as indicated for 15 hours. Medium lactate levels were determined as in C. Metformin treated groups were significantly different from each other. All bar graphs represent mean ± standard deviation. *Indicates significant difference between groups.

In high glucose conditions, metformin treated cells potentially maintain ATP levels by activation of AMPK and enhancing glycolysis [21], [22]. To test this, MCF7 cells were treated with metformin (8 mM) for 15 hours, cellular oxygen consumption (OCR) and extracellular acidification rate (ECAR) were measured using the XF24 seahorse analyzer. As expected, metformin significantly inhibited oxygen consumption of MCF7 cells in medium containing either 25 mM or 2.5 mM glucose (Figure 4A). Enhanced glycolysis in metformin treated cells in 25 mM glucose containing medium was confirmed by increased acidification of the extracellular medium (Figure 4B) and lactate secretion (Figure 4C). However, in low glucose treated cancer cells showed diminished augmentation of ECAR and lactate production by metformin, indicating a reduced ability to promote an increase in glycolysis (Figure 4B, 4C). Thus, failure to sufficiently promote glycolysis correlates with an inability to maintain intracellular ATP under these conditions.

AMPK is known to regulate glycolysis by enhancing glucose uptake and regulation of several key enzymes in the pathway [21]–[25]. Our previous data showed that metformin could activate AMPK by enhancing the phosphorylation of AMPK at Threonine 172 in medium containing 25 mM glucose [14]. Therefore, the levels of AMPK activation with metformin treatment in both high glucose and low glucose were examined. MCF7 and MDAMB231 cells were treated with metformin (8 mM) for one day in either 25 mM or 2.5 mM glucose containing medium. Western blotting was performed to detect phosphorylated AMPK and total AMPK. In both cell lines metformin enhanced the levels of phosphorylated of AMPK, the active form of the enzyme, in medium containing 25 mM glucose, but not in medium containing 2.5 mM glucose after one day treatment (Figure 4D). In contrast, while low glucose, itself, increased phosphorylation of AMPK, the addition of metformin in these conditions appeared to decrease both phospho-AMPK and total levels of the enzyme. This is further demonstrated by densitometery of western blots of MCF7 cells to quantify the ratio of phosphorylated AMPK to total AMPK and the ratio of phosphorylated AMPK to actin (Figure 4D). In medium containing 2.5 mM glucose, metformin still enhanced the activation of AMPK compared to control, demonstrated by the increased ratio of phosphorylated AMPK to total AMPK. However, because of the strong reduction of total AMPK, total phosphorylated AMPK was decreased with metformin treatment compared to control. The reduction of AMPK levels with metformin treatment in medium containing 2.5 mM glucose was associated with significant apoptotic cell death as demonstrated by increased cleaved caspase 7 (Figure 4D lower panel). The variance in levels of active AMPK between high and low glucose containing medium could be the underlying cause of the difference in glycolytic metabolism stimulation by metformin treatment. To further confirm the role of AMPK in promoting glycolysis, MCF7 cells were treated with or without compound C (10 µM), a specific inhibitor of AMPK. After 15 hours in medium containing 25 mM glucose with or without metformin, ECAR as well as lactate measurements were obtained. The extent of ECAR augmentation and accumulation of lactate with metformin treatment was partially blocked by cotreatment with compound C (Figure 4E, 4F). This supports a role for metformin-induced AMPK activation in stimulating glycolysis in high glucose containing medium. In addition, these findings support the conclusion that failure to activate or maintain AMPK activation by metformin in low glucose containing medium leads to depletion of ATP.

**Figure 5:**
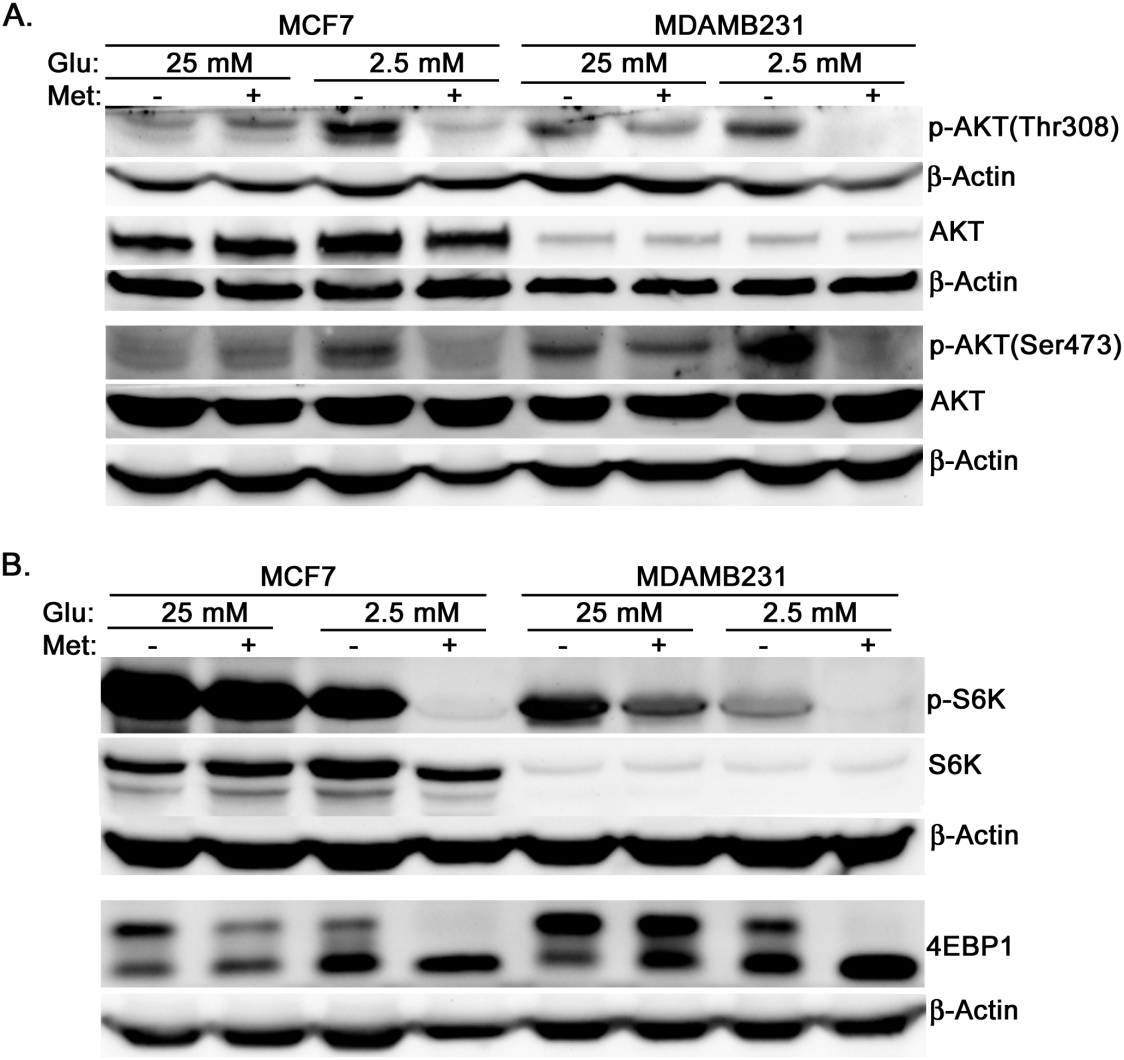
In low glucose medium metformin inhibits AKT phosphorylation and mTOR activation. MCF7 and MDAMB231 cells were treated with metformin in DMEM containing either 25 mM glucose or 2.5 mM glucose for one day. Western blotting was used to estimate the levels of p-AKT (Thr308), p-AKT (Thr473), total AKT, p-S6K, total S6K, 4EBP1 (lower bands represent hypophosphorylated and higher bands represent hyperphosphorylated), and β-actin as loading control.

To better understand the intracellular changes that occur with metformin treatment in high and low glucose media, protein levels of several kinases were examined. Both MCF7 and MDAMB231 cells were treated with metformin (8 mM) for one day in medium containing either 25 mM or 2.5 mM glucose. Western blotting was performed to test phosphorylation of AKT and targets of mTOR signaling. The response to metformin was greatly considerably altered in low glucose conditions. In low glucose conditions metformin was found to substantially reduce the phosphorylation of AKT and decrease the phosphorylation levels of targets of mTOR (S6K and 4EBP1), compared to high glucose conditions (Figure 5A, 5B). Since these pathways are known to promote cell survival as well as glycolytic metabolism, their inhibition by metformin may contribute to reduced glycolysis, subsequent depletion of ATP, and ultimately cell death.

**Figure 6:**
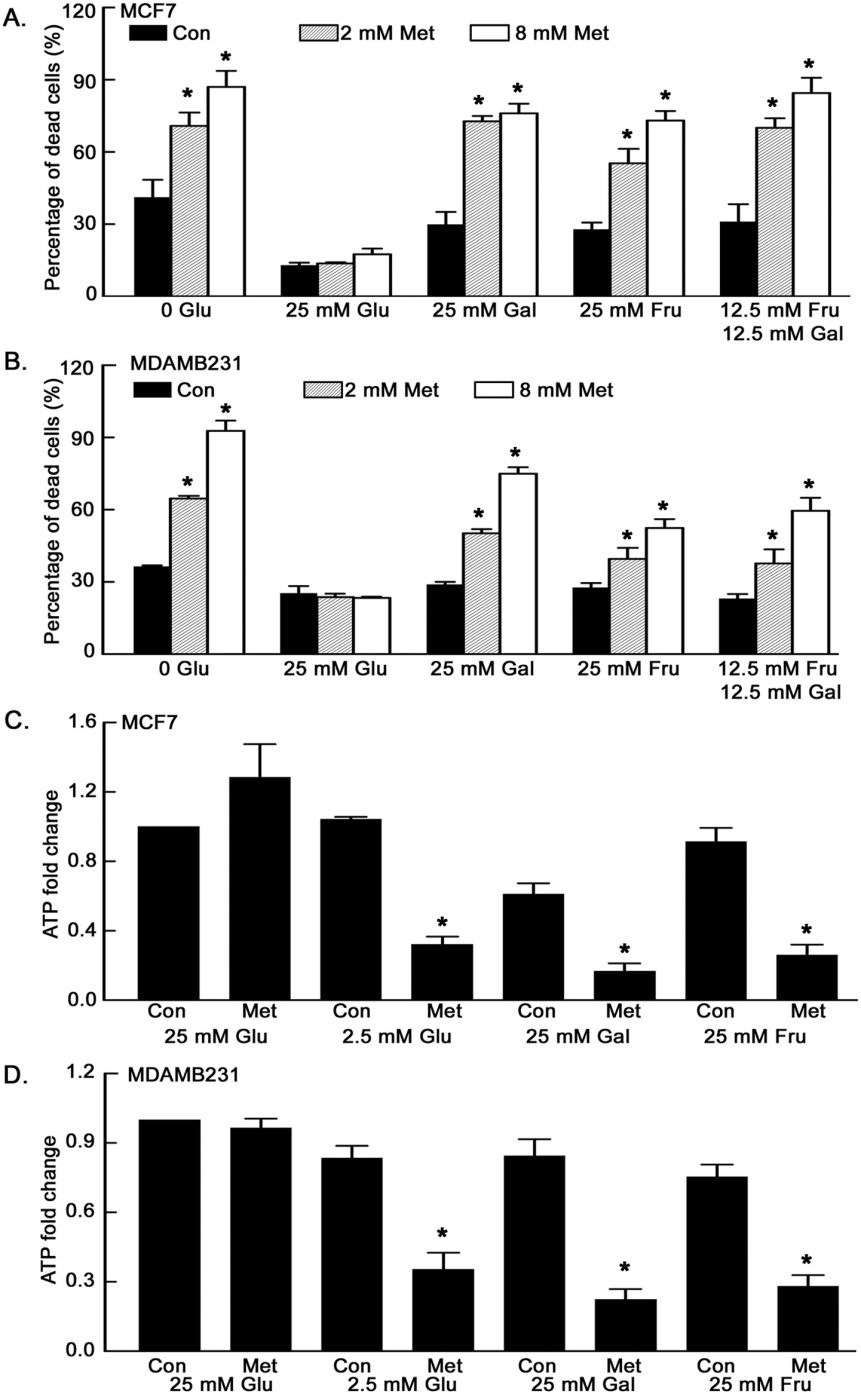
Replacement of glucose with fructose or galactose does not prevent metformin induced cell death or metformin induced ATP reduction. MCF7 (**A**) and MDAMB231 (**B**) cells were treated with metformin in DMEM without glucose, with 25 mM glucose, with 25 mM galactose, 25 mM fructose, or 12.5 mM fructose plus 12.5 mM galactose for 1.5 days. Percentage of dead cells was determined by Sytox Green staining. Metformin treated groups in 25 mM galactose, 25 mM fructose and 12.5 mM fructose plus 12.5 mM galactose were significantly different from metformin treated groups in 25 mM glucose. MCF7 (**C**) and MDAMB231 (**D**) cells were treated in the indicated medium with or without metformin (8 mM) for one day and ATP levels were measured. Metformin treated groups in 2.5 mM glucose, 25 mM galactose and 25 mM fructose were significantly different from their control groups. Results were displayed as fold change compared with control. Data are presented as mean ± standard deviation. *Indicates significant difference between groups.

In order to further investigate the role of glucose in protecting cells from metformin-induced death, the effects of glucose were compared to those of related sugars, including the hexoses fructose and galactose. It has previously been reported that fructose and galactose do not contribute significantly to glycolytic metabolism in cancer cells [26], [27]. Furthermore, processing of galactose through glycolysis results in no net ATP synthesis. Based on these finding we predicted that fructose and galactose would be unable to support ATP synthesis in the presence of metformin. To test this, glucose-free cell culture medium was supplemented with glucose, fructose, or galactose and cultures were again analyzed for cell death (Figure 6A, 6B). Breast cancer cell lines MCF7 and MDAMB231 were treated with or without metformin for 1.5 days in DMEM containing glucose (0 or 25 mM), fructose (25 mM), galactose (25 mM), or fructose plus galactose (12.5 mM each). Similar to previous results, increasing the glucose concentration substantially reduced the cytotoxicity of metformin in both MCF7 and MDAMB231 cells. However, fructose and galactose were not effective in preventing the cytotoxicity of metformin. ATP levels were also examined in cells treated with metformin in medium containing the various hexoses. Metformin treatment led to significantly decreased ATP levels in low glucose, high fructose, or high galactose conditions, but not in high glucose (25 mM) conditions. The reduction in ATP despite high fructose or galactose may explain why these sugars are unable to rescue the cells from metformin induced cytotoxicity.

**Figure 7:**
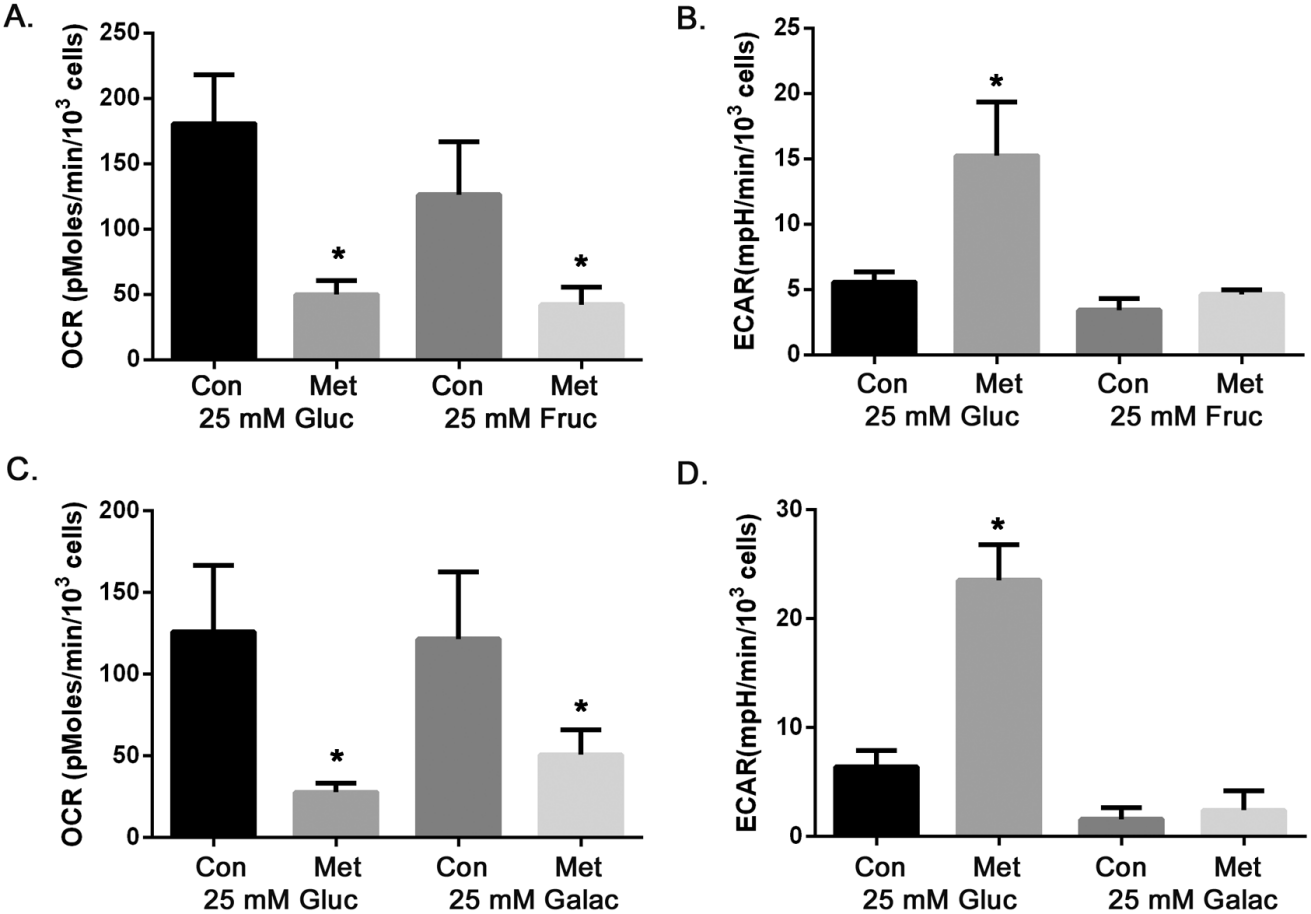
High fructose (25 mM) and galactose (25 mM) do not support metformin enhanced glycolysis as observed in high glucose medium (25 mM). MCF7 cells were treated with metformin (8 mM) in medium containing 25 mM glucose, 25 mM fructose or 25 mM galactose for 15 hours. OCR and ECAR were measured. **A.** OCR in either high glucose or fructose conditions, metformin treated groups were significantly different from their control groups, **B.** ECAR in either high glucose or fructose conditions, metformin treated groups were significantly different from each other, **C.** OCR in either high glucose or galactose containing media, metformin treated groups were significantly different from their control groups, **D.** ECAR in either high glucose or galactose conditions, metformin treated groups were significantly different from each other. Data are presented as mean ± standard deviation. *Indicates significant difference between groups.

To further examine glucose as a specific carbon source for maintaining glycolysis and the production of ATP in the presence of metformin, we next tested the effects of the drug on oxidative phosphorylation and glycolysis by measuring OCR and ECAR in 25 mM glucose, 25 mM fructose, or 25 mM galactose media. Metformin inhibited OCR in all three conditions (Figure 7A, 7C), consistent with its effects on electron transport. However, metformin stimulated glycolysis, as measured by ECAR, only in high glucose. Glycolytic metabolism was reduced with either fructose or galactose alone and was not enhanced by metformin treatment (Figure 7B, 7D). This further confirms the role of enhanced glycolysis in maintaining ATP levels and cell survival after oxidative phosphorylation was inhibited by metformin in high glucose containing medium. Failure to maintain highly activated glycolysis in medium containing low glucose, high fructose or high galactose contributes to the depletion of ATP and eventually to cell death caused by metformin.

**Figure 8:**
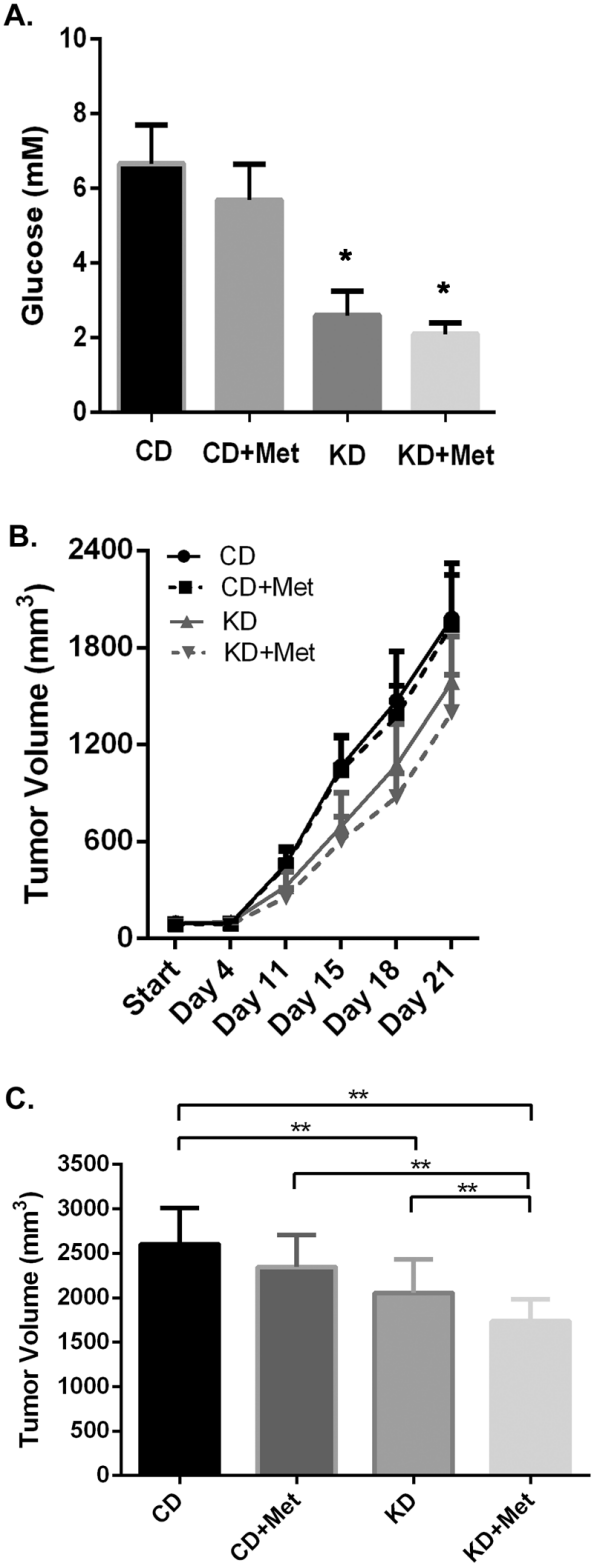
Ketogenic diets reduced serum glucose concentration and enhanced metformin effects on reducing 4T1 breast tumor growth in Balb/c mice. **A.** Serum glucose was measured and demonstrated as bar graph. Data are presented as mean ± standard deviation. Groups on Ketogenic diets are significant different from groups on control diets. **B.** Tumor growth was measured at indicated time for CD (control diet), CD+Met (control diet plus metformin), KD (ketogenic diet) and KD+Met (ketogenic diet plus metformin). CD+Met treatment group was significantly different from KD+Met treatment group after day 11. **C.** Tumor volume at day 23. Data are presented as mean ± standard deviation (**indicates significant difference between groups).

The cell culture experiments described above suggest that limiting the availability of glucose to tumors would enhance the anti-cancer effects of metformin in vivo. To test this a calorie restricted low carbohydrate ketogenic diet was used to lower serum glucose in Balb/c mice. Tumors were established from mouse mammary cancer 4T1 cells and tumor growth was monitored. The low carbohydrate ketogenic diet (KD) significantly reduced serum glucose levels (Figure 8A) from ∼6 mM to below 3 mM. Metformin appeared to induce a slight reduction in serum glucose levels in both diets. Metformin had no effect on tumor growth in mice being fed the control diet (CD). The ketogenic diet alone slowed tumor growth and the slowest tumor growth was observed in mice on the ketogenic diet that were treated with metformin (Figure 8B, 8C). This suggests that lowering glucose in vivo enhances metformin cytotoxicity to cancer cells.

## Discussion

A common criticism of current metformin research is that in vitro concentrations are unattainable *in vivo*. While previous groups have shown that metformin is concentrated within cells, especially the mitochondria [28], [29], the current work also suggests that the use of high glucose media masks some of the effects of metformin, requiring higher doses to mimic the effects observed *in vivo*. While commonly used glucose concentration is 25 mM in cell culture growth media, plasma levels of glucose are usually maintained in a range of 5–7 mM. Using low concentrations of glucose in vitro may also be more relevant to metformin’s mechanism *in vivo*. Using low glucose concentrations can mimic the systemic glucose modulation effects of metformin and potentially provide a more accurate view of the pathways activated within cancer cells.

Our results support and extend previous observations of enhanced effects of glucose deprivation on metformin activity against tumor cells. Several groups have found synergism between metformin or other biguanides and 2-deoxyglucose, a compound that blocks glycolysis and has similar effects as glucose deprivation [16], [30]–[32]. Menendez *et al*. [15] reported increased cell death of breast cancer cells when treated with metformin in the absence of glucose. Our data suggest that similar increases in cell death are observed when glucose is reduced to physiologic levels of metformin and does not require complete elimination of the sugar. Saito et al. [33] also demonstrated that the cytotoxic effects of metformin and other biguanides to cancer cells are dramatically enhanced in glucose-free conditions. This same group went on to show that biguanides inhibit the unfolded protein response (UPR) that is induced by glucose deprivation. They found that this effect was mediated by hyperactivation of 4EBP1 through mTOR inhibition and that this was likely to be independent of AMPK [16]. Our data also show that metformin, in low glucose conditions, causes a dramatic decrease in phosphorylation of 4EBP1 and other mTOR targets, while at the same time failing to increase phosphorylated (active) AMPK. Javeshghani et al. [17] observed that the sensitivity of colon cancer cells to metformin was dependent on the fuel source. They found that the effects of metformin were enhanced by a lack of glucose but not by a lack of glutamine, which requires mitochondrial metabolism for production of ATP. As in our studies, they observed a drop in ATP levels when cells were treated with metformin under glucose deprivation. They also found that metformin could enhance lactate production only in cells cultured in high glucose conditions. Our results confirm these finding and also show that enhanced lactate production in metformin treated cells under high glucose conditions is associated with activation of AMPK. We have also found that the alternative hexose fuels fructose and galactose are unable to prevent metformin cytotoxicity. It is known that neither fructose or galactose contributes significantly to glycolytic metabolism in cancer cells [27]. Furthermore, the AMPK inhibitor compound C reduces the ability of metformin to promote glycolysis. This last observation is consistent with the findings of Shackelford et al. [34] who showed that loss of LKB1, a tumor suppressor that phosphorylates and activates AMPK, selectively enhances the anticancer effects of the biguanide phenformin.

Based on our results and the discussion above we propose that high glucose protects against metformin cytotoxicity by providing a fuel source for glycolytic metabolism, which maintains cellular ATP levels even when metformin blocks mitochondrial oxidative metabolism. Enhanced glycolytic metabolism induced by metformin requires activation of AMPK and the availability of glucose allows glycolytic metabolism to run at high efficiency. When glucose is limiting, AMPK is not effectively activated by metformin and cancer cells lack sufficient fuel to maintain glycolytic metabolism. Also, mTOR signalling is blocked in an AMPK-independent manner, further enhancing the metabolic deficiency. Cellular ATP becomes depleted, leading to energy collapse and cell death. This is consistent with previous reports of the ability of AMPK signalling to maintain ATP levels in response to hypoxia, fuel deprivation, and other stressors [35]. Interestingly, non-cancer cells do not appear to be sensitized to the cytotoxic effects of metformin by glucose deprivation (see Fig. 1D). It is likely that normal cells are less dependent on glucose as a fuel source and are able to maintain ATP levels by utilizing other glycolytic substrates.

Notably, when glucose concentration is lowered, the pH change in media induced by metformin treatment no longer occurs. This is likely a result of less lactate production when normal to low glucose concentrations are used. High lactate production has recently been proposed as a protective factor to glucose deprivation [36]. High glucose concentrations with metformin treatment may have increased lactate production and lactic acidosis in cell culture to levels that do not occur in patients. In type 2 diabetes patients taking metformin, the risk of lactic acidosis in patients with normal renal function is minimal and was found to be no greater than any other anti-hyperglycemic agent [37]. Taken together, these results suggest modifications in glucose concentration *in vitro* may provide a more accurate model of studying cancer, and further highlight the importance of reducing glucose availability to cancer cells. Despite a long history of use as a diabetes medication, the precise cellular actions of metformin are still largely a mystery. Furthermore, the mechanism of action for metformin’s cancer inhibition and killing effects remains unidentified. The results reported in this study contribute to evidence that both the systemic glucose lowering effects and direct cancer cellular effects contribute to metformin’s mechanism of action. The increased cancer cytotoxicity when glucose concentrations were lowered may also be evidence that the multiple pathways affected by metformin enhance one another to promote cancer cell death. As discussed above, alternative carbon energy sources such as fructose and galactose did not alter metformin’s cancer inhibitory and cell death effects in the same manner as increasing glucose concentrations. This suggests that the altered cancer cell metabolism has sacrificed flexibility in carbon source in order to maintain rapid proliferation [26], [27]. Thus, attempting to lower available glucose through methods such as diet may simultaneously enhance metformin treatment and inhibit cancer growth. Zhou et al. showed that calorically restricted ketogenic diet could significantly lower plasma glucose level and was an effective therapeutic alternative for malignant brain tumor [38]. We used a similar commercially available ketogenic diet with calories restriction and successfully obtained serum glucose reduction from ∼6 mM to below 3 mM in Balb/c mice. Mice on a ketogenic diet and metformin treatment showed the slowest tumor growth. This further emphasizes the importance of glucose deprivation in determining metformin cytotoxicity.

Oleksyszyn proposed using a ketogenic diet with metformin as anti-cancer therapy to control glucose levels [39]. This is based on the theory that systemically lowering serum glucose is associated with decreased tumor growth. In that proposal, the focus is the effects of metformin on controlling glucose levels by inhibiting gluconeogenesis rather than direct cytotoxicity to cancer cells. In the mouse model that we have used, metformin only slightly decreases glucose levels in mice on either the control or ketogenic diet. Therefore, the observed tumor growth inhibition with metformin treatment was most likely not due to its effects on glucose regulation but direct cytotoxicity on cancer cells. 4T1 tumor cells are known for their aggressive growth and metastasis in vivo [40]. This limits metformin treatment time before mice have to be euthanized due to emaciation or large tumor burden. This also explains why metformin has not shown effects on slowing tumor growth in mice on control diet with normal serum glucose and limited treatment time. Future studies might be done to evaluate the effects of a ketogenic diet without calorie restriction but with metformin treatment on tumor growth.

These results may also impact clinical use of metformin in cancer patients, as ketogenic diets may be utilized to promote low glucose states while still providing necessary energy for many normal cell types. Ketotic states are common in diets focused on carbohydrate reduction, and fasting states do not appear to be harmful to normal cells. Moreover, metformin and caloric restriction have both been shown to increase lifespan in mice [41] and caloric restriction trials for improved longevity in humans are gaining increased interest. In related studies, Zhu et al. [18] found that metformin combined with dietary energy restriction protected against new tumor occurrence in a rat mammary cancer model.

With metformin’s extensive safety data from decades of use as a diabetic medication, another role for the drug may be as an adjuvant between other courses of therapy such as surgery, radiation, or traditional chemotherapy. In cancers that are difficult to screen and follow such as ovarian cancer, metformin could potentially have use as a relatively low side effect adjuvant therapy following surgical debulking. Because of metformin’s low side effect profile, therapy could potentially be initiated much sooner following surgery and would generally not interfere with the standard chemotherapy schedule. In patients with lower risk stratification, where chemotherapy is generally not indicated, adjuvant therapies such as metformin may be a useful addition to routine surveillance. Metformin could potentially play a similar role in cancers as tamoxifen maintenance therapy in estrogen receptor positive breast cancers. While metformin’s effect may not be as strong as tamoxifen’s, metformin is also not limited by estrogen receptor status. Past metformin research has shown many benefits: from promising epidemiological data for cancer reduction [42] to direct cancer stem cell killing [11]. This research provides additional evidence that metformin has direct cancer cytotoxic effects, and that these effects are enhanced with lower glucose concentrations. Future research will help to better define metformin’s role as a potential cancer therapy.

## References

[1] Sandulache VC, Ow TJ, Pickering CR, Frederick MJ, Zhou G, et al.. (2011) Glucose, not glutamine, is the dominant energy source required for proliferation and survival of head and neck squamous carcinoma cells. Cancer. 10.1002/cncr.25868 PMC3135768 21692052

[2] Han L, Ma Q, Li J, Liu H, Li W, et al (2011) High glucose promotes pancreatic cancer cell proliferation via the induction of EGF expression and transactivation of EGFR. PLoS One 6: e27074. 22087246 10.1371/journal.pone.0027074 PMC3210779

[3] Sinnett-Smith J, Kisfalvi K, Kui R, Rozengurt E (2013) Metformin inhibition of mTORC1 activation, DNA synthesis and proliferation in pancreatic cancer cells: dependence on glucose concentration and role of AMPK. Biochem Biophys Res Commun 430: 352–357. 23159620 10.1016/j.bbrc.2012.11.010 PMC3545113

[4] De Lorenzo MS, Baljinnyam E, Vatner DE, Abarzua P, Vatner SF, et al (2011) Caloric restriction reduces growth of mammary tumors and metastases. Carcinogenesis 32: 1381–1387. 21665891 10.1093/carcin/bgr107 PMC3165123

[5] Lee C, Longo VD (2011) Fasting vs dietary restriction in cellular protection and cancer treatment: from model organisms to patients. Oncogene 30: 3305–3316. 21516129 10.1038/onc.2011.91

[6] Seyfried TN, Kiebish MA, Marsh J, Shelton LM, Huysentruyt LC, et al (2011) Metabolic management of brain cancer. Biochim Biophys Acta 1807: 577–594. 20804725 10.1016/j.bbabio.2010.08.009

[7] DeFronzo RA, Goodman AM (1995) Efficacy of metformin in patients with non-insulin-dependent diabetes mellitus. The Multicenter Metformin Study Group. N Engl J Med 333: 541–549. 7623902 10.1056/NEJM199508313330902

[8] Rizkalla SW, Elgrably F, Tchobroutsky G, Slama G (1986) Effects of metformin treatment on erythrocyte insulin binding in normal weight subjects, in obese non diabetic subjects, in type 1 and type 2 diabetic patients. Diabete Metab 12: 219–224. 3533670

[9] Knowler WC, Barrett-Connor E, Fowler SE, Hamman RF, Lachin JM, et al (2002) Reduction in the incidence of type 2 diabetes with lifestyle intervention or metformin. N Engl J Med 346: 393–403. 11832527 10.1056/NEJMoa012512 PMC1370926

[10] Gotlieb WH, Saumet J, Beauchamp MC, Gu J, Lau S, et al (2008) In vitro metformin anti-neoplastic activity in epithelial ovarian cancer. Gynecol Oncol 110: 246–250. 18495226 10.1016/j.ygyno.2008.04.008

[11] Hirsch HA, Iliopoulos D, Tsichlis PN, Struhl K (2009) Metformin selectively targets cancer stem cells, and acts together with chemotherapy to block tumor growth and prolong remission. Cancer Res 69: 7507–7511. 19752085 10.1158/0008-5472.CAN-09-2994 PMC2756324

[12] Buzzai M, Jones RG, Amaravadi RK, Lum JJ, DeBerardinis RJ, et al (2007) Systemic treatment with the antidiabetic drug metformin selectively impairs p53-deficient tumor cell growth. Cancer Res 67: 6745–6752. 17638885 10.1158/0008-5472.CAN-06-4447

[13] Vazquez-Martin A, Oliveras-Ferraros C, Menendez JA (2009) The antidiabetic drug metformin suppresses HER2 (erbB-2) oncoprotein overexpression via inhibition of the mTOR effector p70S6K1 in human breast carcinoma cells. Cell Cycle 8: 88–96. 19106626 10.4161/cc.8.1.7499

[14] Zhuang Y, Miskimins WK (2008) Cell cycle arrest in Metformin treated breast cancer cells involves activation of AMPK, downregulation of cyclin D1, and requires p27Kip1 or p21Cip1. J Mol Signal 3: 18. 19046439 10.1186/1750-2187-3-18 PMC2613390

[15] Menendez JA, Oliveras-Ferraros C, Cufi S, Corominas-Faja B, Joven J, et al (2012) Metformin is synthetically lethal with glucose withdrawal in cancer cells. Cell Cycle 11: 2782–2792. 22809961 10.4161/cc.20948

[16] Matsuo J, Tsukumo Y, Saito S, Tsukahara S, Sakurai J, et al (2012) Hyperactivation of 4E-binding protein 1 as a mediator of biguanide-induced cytotoxicity during glucose deprivation. Mol Cancer Ther 11: 1082–1091. 22402126 10.1158/1535-7163.MCT-11-0871

[17] Javeshghani S, Zakikhani M, Austin S, Bazile M, Blouin MJ, et al (2012) Carbon source and myc expression influence the antiproliferative actions of metformin. Cancer Res 72: 6257–6267. 23041548 10.1158/0008-5472.CAN-12-2907

[18] Zhu Z, Jiang W, Thompson MD, McGinley JN, Thompson HJ (2011) Metformin as an energy restriction mimetic agent for breast cancer prevention. J Carcinog 10: 17. 21799661 10.4103/1477-3163.83043 PMC3142764

[19] Zhuang Y, Miskimins WK (2011) Metformin induces both caspase-dependent and poly(ADP-ribose) polymerase-dependent cell death in breast cancer cells. Mol Cancer Res 9: 603–615. 21422199 10.1158/1541-7786.MCR-10-0343 PMC3096726

[20] Batandier C, Guigas B, Detaille D, El-Mir MY, Fontaine E, et al (2006) The ROS production induced by a reverse-electron flux at respiratory-chain complex 1 is hampered by metformin. J Bioenerg Biomembr 38: 33–42. 16732470 10.1007/s10863-006-9003-8

[21] Marsin AS, Bertrand L, Rider MH, Deprez J, Beauloye C, et al (2000) Phosphorylation and activation of heart PFK-2 by AMPK has a role in the stimulation of glycolysis during ischaemia. Curr Biol 10: 1247–1255. 11069105 10.1016/s0960-9822(00)00742-9

[22] Wu SB, Wei YH (2012) AMPK-mediated increase of glycolysis as an adaptive response to oxidative stress in human cells: implication of the cell survival in mitochondrial diseases. Biochim Biophys Acta 1822: 233–247. 22001850 10.1016/j.bbadis.2011.09.014

[23] Andrade BM, Cazarin J, Zancan P, Carvalho DP (2012) AMP-activated protein kinase upregulates glucose uptake in thyroid PCCL3 cells independent of thyrotropin. Thyroid 22: 1063–1068. 22953992 10.1089/thy.2012.0041

[24] Habibollahi P, van den Berg NS, Kuruppu D, Loda M, Mahmood U (2013) Metformin–an adjunct antineoplastic therapy–divergently modulates tumor metabolism and proliferation, interfering with early response prediction by 18F-FDG PET imaging. J Nucl Med 54: 252–258. 23376854 10.2967/jnumed.112.107011 PMC3703242

[25] Smith TA, Zanda M, Fleming IN (2013) Hypoxia stimulates 18F-fluorodeoxyglucose uptake in breast cancer cells via hypoxia inducible factor-1 and AMP-activated protein kinase. Nucl Med Biol 40: 858–864. 23786679 10.1016/j.nucmedbio.2013.05.006

[26] Marroquin LD, Hynes J, Dykens JA, Jamieson JD, Will Y (2007) Circumventing the Crabtree effect: replacing media glucose with galactose increases susceptibility of HepG2 cells to mitochondrial toxicants. Toxicol Sci 97: 539–547. 17361016 10.1093/toxsci/kfm052

[27] Reitzer LJ, Wice BM, Kennell D (1979) Evidence that glutamine, not sugar, is the major energy source for cultured HeLa cells. J Biol Chem 254: 2669–2676. 429309

[28] El-Mir MY, Nogueira V, Fontaine E, Averet N, Rigoulet M, et al (2000) Dimethylbiguanide inhibits cell respiration via an indirect effect targeted on the respiratory chain complex I. J Biol Chem. 275: 223–228. 10.1074/jbc.275.1.223 10617608

[29] Owen MR, Doran E, Halestrap AP (2000) Evidence that metformin exerts its anti-diabetic effects through inhibition of complex 1 of the mitochondrial respiratory chain. Biochem J 348 Pt 3: 607–614. PMC1221104 10839993

[30] Ben Sahra I, Laurent K, Giuliano S, Larbret F, Ponzio G, et al (2010) Targeting cancer cell metabolism: the combination of metformin and 2-deoxyglucose induces p53-dependent apoptosis in prostate cancer cells. Cancer Res 70: 2465–2475. 20215500 10.1158/0008-5472.CAN-09-2782

[31] Sandulache VC, Ow TJ, Pickering CR, Frederick MJ, Zhou G, et al (2011) Glucose, not glutamine, is the dominant energy source required for proliferation and survival of head and neck squamous carcinoma cells. Cancer 117: 2926–2938. 21692052 10.1002/cncr.25868 PMC3135768

[32] Lea MA, Chacko J, Bolikal S, Hong JY, Chung R, et al (2011) Addition of 2-deoxyglucose enhances growth inhibition but reverses acidification in colon cancer cells treated with phenformin. Anticancer Res 31: 421–426. 21378320

[33] Saito S, Furuno A, Sakurai J, Sakamoto A, Park HR, et al (2009) Chemical genomics identifies the unfolded protein response as a target for selective cancer cell killing during glucose deprivation. Cancer Res 69: 4225–4234. 19435925 10.1158/0008-5472.CAN-08-2689

[34] Shackelford DB, Abt E, Gerken L, Vasquez DS, Seki A, et al (2013) LKB1 inactivation dictates therapeutic response of non-small cell lung cancer to the metabolism drug phenformin. Cancer Cell 23: 143–158. 23352126 10.1016/j.ccr.2012.12.008 PMC3579627

[35] Bonini MG, Gantner BN (2013) The multifaceted activities of AMPK in tumor progression–why the “one size fits all” definition does not fit at all? IUBMB Life 65: 889–896. 24265196 10.1002/iub.1213

[36] Wu H, Ding Z, Hu D, Sun F, Dai C, et al.. (2011) Central role of lactic acidosis in cancer cell resistance to glucose deprivation-induced cell death. J Pathol. 10.1002/path.3978 22190257

[37] Salpeter SR, Greyber E, Pasternak GA, Salpeter EE (2010) Risk of fatal and nonfatal lactic acidosis with metformin use in type 2 diabetes mellitus. Cochrane Database Syst Rev: CD002967. 10.1002/14651858.CD002967.pub3 20091535

[38] Zhou W, Mukherjee P, Kiebish MA, Markis WT, Mantis JG, et al (2007) The calorically restricted ketogenic diet, an effective alternative therapy for malignant brain cancer. Nutr Metab (Lond) 4: 5. 17313687 10.1186/1743-7075-4-5 PMC1819381

[39] Oleksyszyn J (2011) The complete control of glucose level utilizing the composition of ketogenic diet with the gluconeogenesis inhibitor, the anti-diabetic drug metformin, as a potential anti-cancer therapy. Med Hypotheses 77: 171–173. 21530093 10.1016/j.mehy.2011.04.001

[40] Aslakson CJ, Miller FR (1992) Selective events in the metastatic process defined by analysis of the sequential dissemination of subpopulations of a mouse mammary tumor. Cancer Res 52: 1399–1405. 1540948

[41] Martin-Montalvo A, Mercken EM, Mitchell SJ, Palacios HH, Mote PL, et al (2013) Metformin improves healthspan and lifespan in mice. Nat Commun 4: 2192. 23900241 10.1038/ncomms3192 PMC3736576

[42] Evans JM, Donnelly LA, Emslie-Smith AM, Alessi DR, Morris AD (2005) Metformin and reduced risk of cancer in diabetic patients. BMJ 330: 1304–1305. 15849206 10.1136/bmj.38415.708634.F7 PMC558205

